# Characteristics of Organ Donors Who Died From Suicide by Hanging in Australia and New Zealand: A Retrospective Study

**DOI:** 10.7759/cureus.19243

**Published:** 2021-11-03

**Authors:** Mohamed Fayed, Raju Pusapati, Neil Widdicombe, Matthew Sypek, Rowaa Ibrahim, Nicholas Yeldo, Donald H Penning

**Affiliations:** 1 Anesthesiology, Henry Ford Health System, Detroit, USA; 2 Intensive Care Unit, Queensland Health, Hervey Bay, AUS; 3 Intensive Care Unit, Queensland Health, Brisbane, AUS; 4 Nephrology, Royal Melbourne Hospital, Melbourne, AUS

**Keywords:** solid organ transplant, suicide rates, critical care, team based end of life care, end of life, organ donation, brain death organ management, brain death

## Abstract

Background and objective

The annual incidence of suicide by hanging in Australia and New Zealand has increased in the past decade, and a significant number of these individuals are becoming organ donors. The rates of organ donation following deaths from hanging is unknown and the characteristics of this cohort of donors have not been described in the literature. In light of this, we aimed to examine the trends in organ donation from individuals who had died from hanging, based on the solid organ donor data from the Australia and New Zealand Organ Donation (ANZOD) Registry.

Methods

We conducted a retrospective study that analyzed the ANZOD Registry donor data (2006-2015) to describe the characteristics of solid organ donors who had died by hanging (post-hanging group); these characteristics were compared to those of individuals who died by all other causes (non-hanging group).

Results

During the study period, the number and proportion of donors who died by suicide from hanging increased. Of the 4,024 consented organ donors, 226 had died by hanging and 3,798 had died from other causes. The probability that an individual who died by hanging would become an organ donor increased from 0.5 to 3%. Compared to donors who died by all other causes, post-hanging donors were younger (median age of 30 vs. 50 years), with fewer comorbidities, and a higher incidence of smoking. There was no significant difference in the proportion of those who indicated a prior intent to donate organs between post-hanging (34%) and non-hanging donors (38%). A higher proportion of post-hanging donors donated via the donation after the circulatory death pathway (36.3%) than non-hanging donors (24.2%). Individuals in the post-hanging cohort donated an average of 4.19 organs compared to 3.62 in the non-hanging cohort.

Conclusion

We believe the findings of this retrospective analysis will help inform clinical decision-making regarding organ donation, including the best approaches to obtaining donation consent. Our findings will help physicians provide care to patients and to families of individuals in this challenging group, where organ donation potential is high. Further investigations are required to determine which aspects of healthcare influence the donation rates in individuals who have died by hanging and the outcomes related to transplanted organs.

## Introduction

In industrialized nations, suicide has become one of the leading causes of death among young adults, and it is rising at an alarming rate [1,2]. In 2017, 3,126 people died from intentional self-harm in Australia, representing an increase of 9% from 2016 [3]. The rates of suicide by hanging have been rising compared to other modes of suicide [2,4], with 58% of suicides occurring from hanging in 2017 [3], an increase of 3% from the previous year [5]. However, the incidence of suicide among indigenous Australians might still be underestimated [6].

Anecdotal evidence from the past 10 years suggests that the number of individuals who donated organs after having died from suicide by hanging has increased significantly. However, there is scant data in the literature describing the characteristics of this cohort of organ donors in Australia and New Zealand. Published data in this area have mainly comprised small case series or have focused on transplantation outcomes in organ recipients from donors who had died by hanging or ligature asphyxia [7,8].

In this study, we retrospectively reviewed the characteristics of intended and actual organ donors who had died by hanging over a period of 10 years (2006-2015) and compared them to the general donor cohorts in Australia and New Zealand. To our knowledge, this is the largest Australia- and New Zealand-based study analyzing this cohort with the aim of examining trends in the incidence of organ donation following hanging and describing the demographic characteristics, the donation pathway, and the number of organs retrieved from this group relative to the general donor population. Understanding the variability in potential organ donor populations is important for assessing donation and transplantation outcomes over time and between jurisdictions.

## Materials and methods

Study design

The study design involved a retrospective review of the organ donor data from the Australia and New Zealand Organ Donation (ANZOD) Registry. Donors were divided into two groups according to the cause of death: a “post-hanging group” of donors with hanging recorded as the cause of death and a “non-hanging group” of donors who died from all other causes.

Data sources

ANZOD Registry donor data from January 2006 to January 2015 were collected. Australian Bureau of Statistics mortality data and the New Zealand coronial data were used to determine the total number of deaths caused by hanging, which would serve as the denominator and determine the actual proportion of organ donors.

Variables examined in this study

The following variables were analyzed from the data recorded within the ANZOD Registry:

Incidence and Likelihood of Organ Donation Following Hanging

An “intended donor” was defined as a donor for whom family consent had been documented and blood had been drawn for tissue typing. An “actual donor” was defined as a donor who proceeded to the operating theater for organ retrieval, regardless of whether the organs were successfully used. The incidence and likelihood of organ donation following hanging was trended over the study period.

Patient Demographics

Data related to donor age, gender, and comorbidities, including any history of hypertension, type 2 diabetes, and smoking, were collected.

Prior Intent to Donate

Details of voluntary enrollment in the organ donation registry or notification on the driver’s license were assessed.

Pathway to Donation

Two donation pathways were analyzed: donation after brain death (DBD) and donation after cardiac death (DCD).

Organ Retrieval

The number and types of organs (lung, heart, liver, and kidney) donated were analyzed. For the purposes of organ counting, the following ANZOD rules were applied: each organ was considered a separate organ for counting purposes even if transplanted into the same recipient (e.g., double lung transplant = two organs; en bloc double kidney transplant = two organs). Organs were considered as a single organ even when divided between two recipients (e.g., split livers = one organ). Tissues (e.g., corneas, islet cells, and hepatocytes) were not considered organs.

Donor characteristics of individuals in the “post-hanging group” and those in the “non-hanging group” were compared. The age distribution was assessed for normality using graphical methods (histogram) and numerical methods (Shapiro-Wilk test). This continuous variable was not normally distributed and was therefore reported as median with interquartile range. Differences between groups were tested using the Wilcoxon rank-sum test. Categorical variables were expressed as frequencies and percentages and compared with the chi-square test. Differences in proportions across time periods were tested using a two-sample test of proportions (Z score). Differences in the mean number of organs retrieved per donor were compared using a two-sample t-test. We used the threshold p-value ≤0.05 to determine statistical significance. All analyses were conducted using Stata version 14 (StataCorp, College Station, TX).

Funding and ethical consideration

As the project was deemed compliant with the National Health and Medical Research Council of Australia guidance for “ethical considerations in quality assurance and evaluation activities,” Human Resource Ethics Committee review was not required in Australia (Royal Brisbane and Women’s Hospital Ethics Committee, ref: HREC/17/QRBW/175). In New Zealand, the Chief Coroner had approved the release of information under the Official Information Act 1982.

## Results

**Figure 1:**
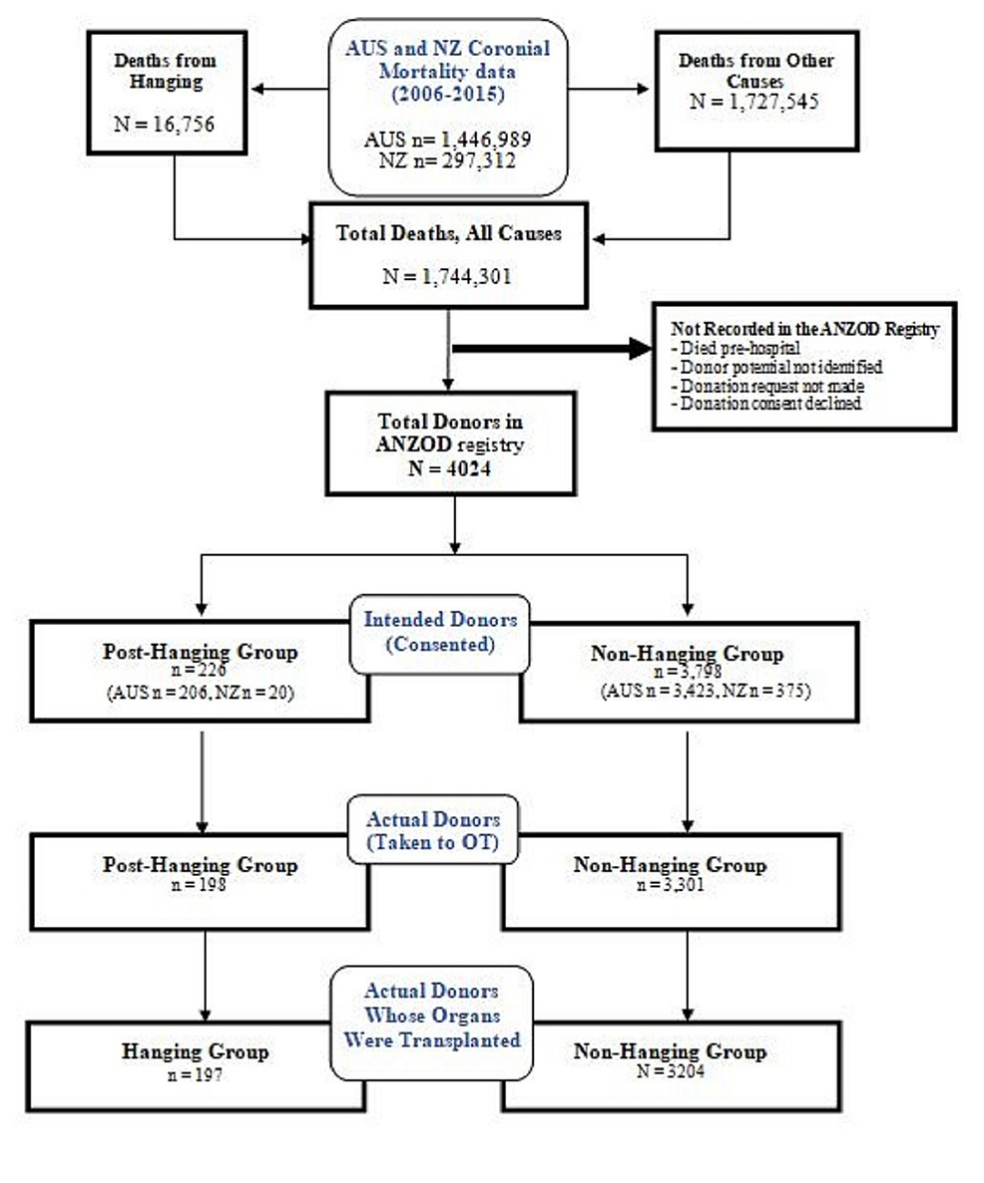
Australia and New Zealand mortality data and ANZOD Registry data 2006-15 AUS: Australia; NZ: New Zealand; ANZOD: Australia and New Zealand Organ Donor; OT: operating theatre

During the 10-year study period, the ANZOD Registry documented a total of 4,024 consented organ donors. In this group, 226 donors had died from hanging, and 3,798 donors had died from other causes (Figure 1).

Incidence and probability of organ donation following deaths from hanging

**Figure 2:**
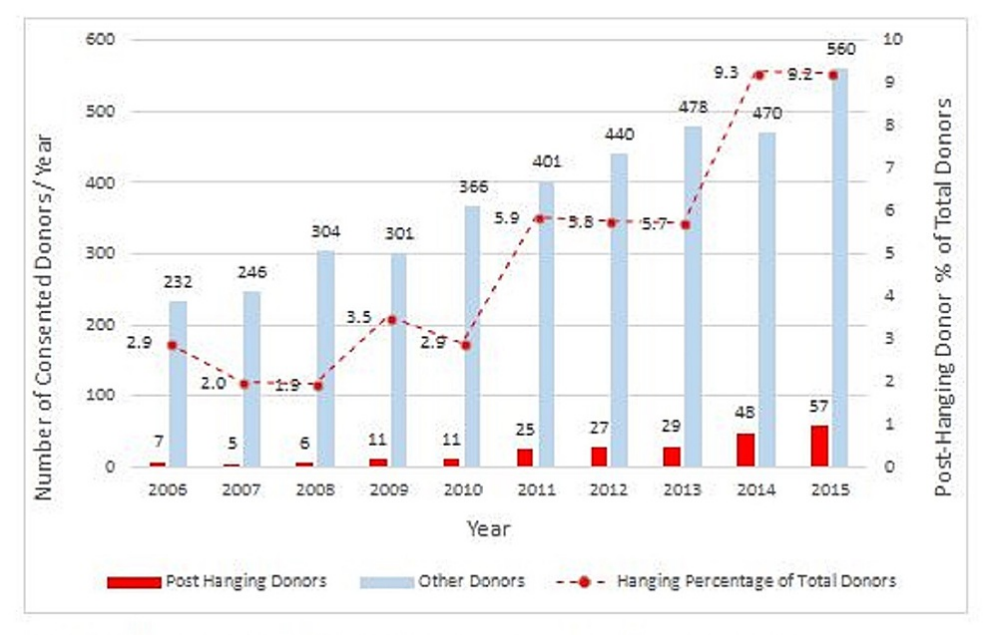
Relative yearly representation of consented donors in each group 2006-15

During the study period, the post-hanging cohort represented 5.62% of the total consented organ donor population and 5.66% of the actual donor population. Over the 10-year period, there was a threefold increase in the proportional representation of post-hanging donors who constituted only 2.93% (7/239) of total donors in 2006/2007. The rate rose steadily to 9.24% (57/617) by 2014/2015 (difference in proportions: 6.3%; 95% CI: 4.6-9.0; p<0.001) (Figure 2).

**Figure 3:**
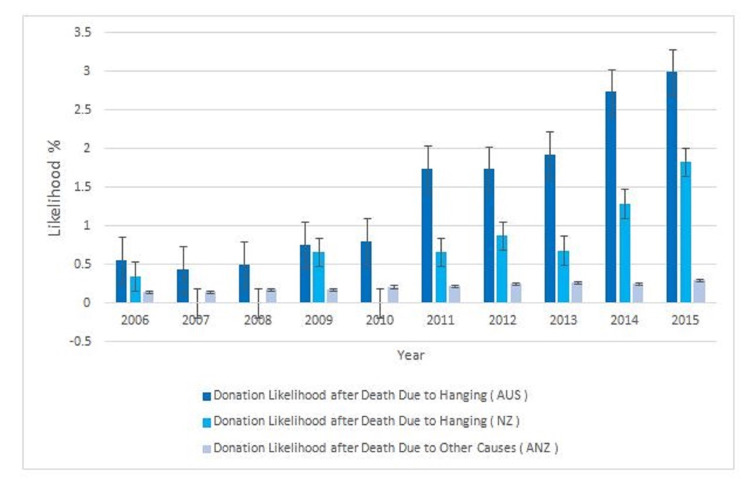
Donation likelihood

Figure 3 illustrates the likelihood of donation after death in both cohorts. 

**Figure 4:**
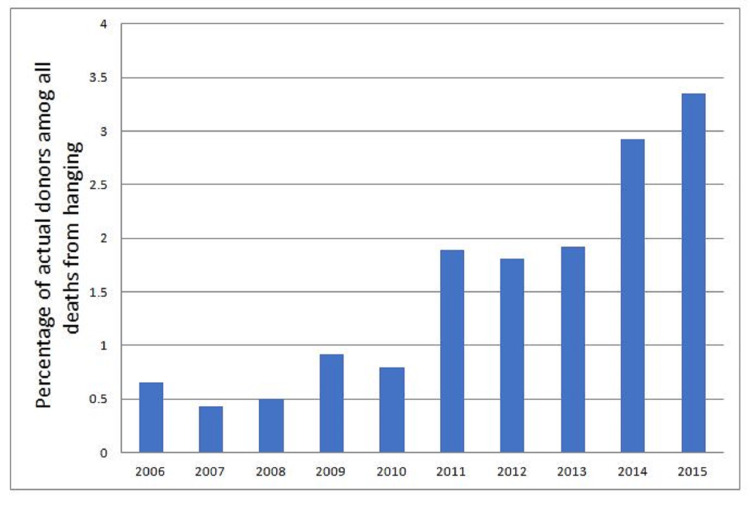
Annual trends in the percentage of actual donors among deaths from hanging

The percentage of actual donors among all deaths from hanging had increased from 0.65% in 2006 to 3.35% in 2015 (Figure 4).

Donor characteristics

Demographics, comorbidities, and donation pathway among consented organ donors in each group are shown in Table 1.

**Table 1:** Characteristics of consented donors in each group DBD: donation after brain death; DCD: donation after cardiac death; IQR: interquartile range

Donor characteristic		Post-hanging (n=226)	Non-hanging (n=3,798)	P-value
Age in years, median (IQR)		30 (23-41)	50 (35-61)	<0.001
Sex, n (%)	Female	91 (40.3%)	1,655 (43.6)	0.33
	Male	135 (59.7%)	2,143 (56.4%)	
Hypertension history, n (%)	No	212 (93.8%)	2,685 (70.7%)	<0.001
	Yes	13 (5.8%)	1,066 (28.1%)	
	Unknown	1 (0.4%)	47 (1.2%)	
Type 2 diabetes, n (%)	No	220 (97.3%)	3,442 (90.6%)	0.001
	Yes	4 (1.8%)	281 (7.4%)	
	Unknown	2 (0.9%)	75 (2%)	
Smoking history, n (%)	Current	132 (58.4%)	1,442 (38.0%)	<0.001
	Former	25 (11.1%)	881 (23.2%)	
	Never	67 (29.6%)	1,452 (38.2%)	
	Unknown	2 (0.9%)	23 (0.6%)	
Donation pathway, n (%)	DBD	144 (63.7%)	2,878 (75.8%)	<0.001
	DCD	82 (36.3%)	919 (24.2%)	
	Unknown	0	1 (<1%)	

Donors in the post-hanging group were significantly younger than those in the non-hanging group (median age of 30 vs. 50 years, p<0.001). Most donors in the post-hanging group were aged 26-35 years, while most donors in the non-hanging group were >56 years in age. There was no significant difference in gender distribution between the two groups. The post-hanging group showed a lower incidence of hypertension and type 2 diabetes, but a higher incidence of active smoking, all of which were significantly different from the non-hanging cohort.

Donation pathway

Over one-third (36.3%) of donors from the post-hanging group donated via the DCD pathway throughout the study period; in contrast, less than one-quarter (24.2%) of the donors in the non-hanging group donated via the DCD pathway over the study period (Table 1).

Prior intent to donate

**Figure 5:**
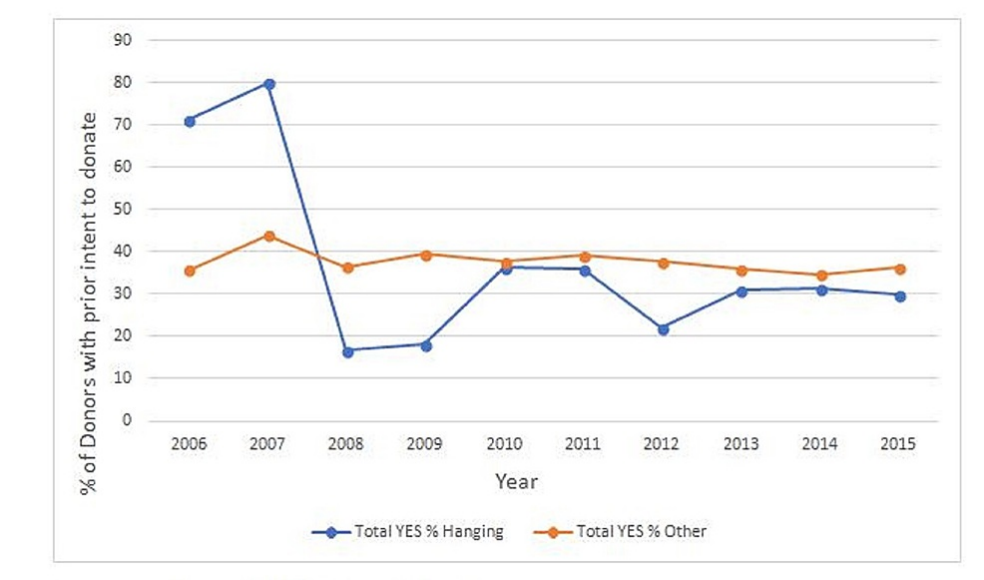
Percentage of donors with prior intent to donate

**Figure 6:**
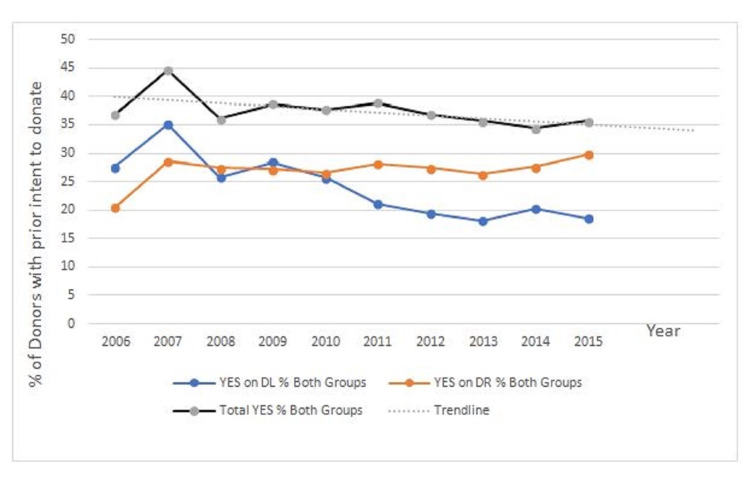
Percentage of donors with prior intent to donate (with trendline)

No significant difference was observed between the post-hanging group and the non-hanging group in the proportion of consented donors who had recorded a prior intent to donate (difference in proportion: 5.1%; 95% CI: -1.2%-11%; p=0.12). Analysis of intent to donate by time periods also did not demonstrate any significant change in registration of prior intent to donate, although a trend toward decreased intent over the duration of the study period was seen, especially in the post-hanging group (Figures 5, 6). There was a significant drop in prior intent to donate from 2007 to 2008, and this was due to the low number of subjects in those years: in 2007, there were five cases, while in 2008, there were six cases.

Organ retrieval

Of note, 88% of the consented organ donors (198/226) from the post-hanging group proceeded to the operating theater and completed an actual organ donation, and 87% of the consented donors (3,301/3,798) from the non-hanging group proceeded to organ donation. Regarding the average number of organs per donor by cause of death, individuals in the post-hanging group donated 4.2 organs on average while those in the non-hanging group donated 3.6 organs on average (difference: 0.57 organs per donor; 95% CI: 0.32-0.81; p<0.001). However, when looking at the number of organs donated by age category, no statistically significant difference between the two groups was observed (Table 2).

**Table 2:** Mean number of organs donated by age group

Age category (years)	Mean organs donated		P-value
	Post-hanging	Non-hanging	
0-15	4.81	3.99	0.593
16-25	4.41	4.67	0.282
26-35	4.23	4.27	0.888
36-45	4.30	4.04	0.341
46-55	3.39	3.59	0.535
56+	2.75	2.86	0.813

**Figure 7:**
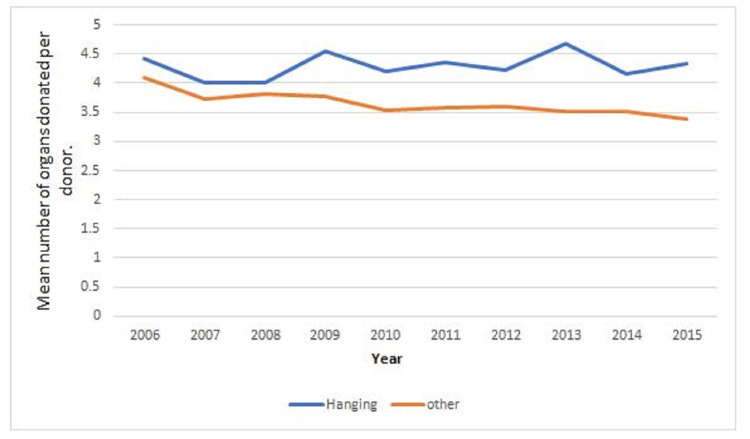
Mean number of organs donated over time

There was no clear trend over the study period in the post-hanging group, with an average of 4.4 organs donated in 2006 and 4.3 organs in 2015 (Figure 7). In contrast, there was a downtrend in organs retrieved per donor in the other groups from 2006 to 2015.

Regarding the proportion of donors from whom specific organs were retrieved, individuals from the post-hanging group were more likely to be multiple organ donors. With regard to specific organs, more lungs were retrieved proportionally (53% vs. 38%) as well as more heart (31% vs. 24%) and more whole livers (56% vs. 54%) from the post-hanging group compared to the non-hanging group, respectively.

## Discussion

In this study, we demonstrated an increasing trend in the number of individuals who died from suicide by hanging and a rise in the likelihood that those individuals had been solid organ donors. The proportion of death from self-harm increased from 1.6 to 2% of all-cause mortality over the study period. In Australia, 1,080 deaths occurred from suicidal hanging in 2006 [9], which increased to 1,705 in 2015, representing 56% of total deaths from suicide. There have also been significant changes in organ donation practices and resourcing with an associated increase in organ donation rates in the past decade.

The increase in the number of individuals who have died from suicide by hanging has been attributed to the drop in suicide rates due to other causes, such as firearms [2]. Other factors such as improvements in both prehospital and critical care management have led to more post-hanging patients surviving to provide organ donation. The implementation of a number of task force recommendations, including a standardized donation process in hospitals combined with a proactive donor detection and audit program performed by well-trained transplant coordinators [10], may also account for the increasing proportional representation of this group of individuals among the consented organ donors registered in the ANZOD Registry. Although we did not study the survival rate in terms of hospital admissions or in-hospital mortality following attempted suicidal hanging, previous studies have shown that only 22% of individuals who had hanged themselves were transferred alive to emergency departments [11], and the in-hospital mortality was 23% [12]. Using this to approximate the in-hospital mortality rate among the 16,756 persons who died from hanging during our study period yields an estimated figure of 848 patients who may have been considered potential donors. The 226 consented donors in the post-hanging group, as identified in the ANZOD data, would therefore constitute only 27% of such potential donors following hanging. This suggests that the detection, requesting, and consenting rate of potential donors following hanging may be much lower than the overall rate of 54-59% per national performance data published annually by the Australian Organ and Tissue Authority [13].

Knowledge of an individual’s prior intent regarding organ donation is the most important factor associated with family consent [14]. Our study did not demonstrate a difference in the rates of pre-existing donation intent between the two groups as recorded in the ANZOD. However, another study has shown that a prior intent to donate is higher in individuals who died from suicide [15].

We examined a history of comorbidities such as hypertension, type 2 diabetes, and smoking in our cohorts, as these are the commonly recorded (and easily validated) comorbidities that can impact medical suitability for organ donation. Our study showed that organ donors who had died by hanging were younger and had fewer comorbidities, but had a higher incidence of smoking compared to other donor groups. This correlates with some previous studies [8,16,17], although some other studies showed no significant difference [18,19].

Relative to donors dying from other causes, a greater proportion of donors in the post-hanging group donated via the DCD pathway. However, this did not change over time, making it unlikely that increased clinician uptake of DCD programs accounted for the increase in the number of post-hanging organ donors. While such patients may have met the “GIVE trigger” for identifying potential donors, neuro-prognostication in a young person sustaining severe hypoxic-ischemic encephalopathy from attempted suicide may have influenced an early clinical decision to recommend a palliative course, rather than pursue a formal diagnosis of brain death. There is also an association between hanging and cervical spine injury [20], confounding apnea testing for a formal diagnosis of brain death. This is especially significant in regional Australia, where 4-vessel cerebral angiography and other diagnostics may not be available to confirm the diagnosis of brain death in these situations. This explains why providers pursue the DCD pathway rather than the DBD pathway more often in post-hanging patients.

We found that donors who had died from hanging had a higher mean number of organs retrieved per donor compared to the other donor cohort. This higher number was consistent with various organs in both groups. Donated organ types were similar between the two groups. There are reservations regarding the medical suitability of organ retrieval from hanging victims based on risks of global hypoxia that often occurs in hanging and the resultant warm ischemic injury that can potentially affect the transplantable organs. These factors are especially pertinent during donation via the DCD pathway. The medical suitability of lung donation is particularly controversial because individuals who have died from hanging can experience a variety of respiratory injuries (e.g., aspiration and negative pressure pulmonary edema) [21,22]. Conversely, there is a reason to not exclude them from being considered as potential donors, as such patients are younger and have fewer comorbidities. The current published evidence regarding the medical suitability of organ transplantation from individuals who have died from hanging and recipient outcomes from such transplantation is limited and conflicting. There was no difference in outcomes [6,18] or in chronic allograft rejection, but there was a higher incidence of extracorporeal life support [16,17]. One study showed inferior transplant outcomes following lung transplantation with organs from those who died from hanging, but good outcomes following kidney and liver transplantation [8]. And another study showed no difference in outcomes from liver transplantation [19]. Despite these reservations, lung retrieval in our post-hanging donor cohort occurred at a higher rate, notwithstanding a higher percentage of current smokers in this cohort relative to the other donor cohort.

Limitations

Observed changes may represent changes in documentation and data collection rather than actual changes in rates of donation after death caused by hanging. Investigators were unable to identify any specific system change impacting the data collection methodology, other than the establishment of the Australian Organ and Tissue Authority in 2009 and the electronic donor record in 2014. This study was unable to measure the true donation rates among those who died by near-hanging and those who survived to be admitted to ICUs, as this would require prehospital data from the ambulance services, trauma registry, etc. Moreover, some data might not have been recorded because suicide victims might not be able to reach the hospital and hence are not documented in hospital records. Additionally, this study did not examine recipient outcomes over the short and long run. Further studies are needed to evaluate organ transplant outcomes in both groups.

## Conclusions

Gaining awareness about the changes in the solid organ donor population over time has helped to identify an emerging group of donors following death due to hanging. The likelihood that a given person who died from hanging eventually became an actual organ donor has increased over the study period to significantly exceed the average donation likelihood following death due to other causes, making this a distinct donor cohort (such as traumatic brain injury, intracranial hemorrhage, etc.).

As patients in the post-hanging group tended to be younger with fewer comorbidities, there is a high organ donation potential in this group, with such patients donating more organs per donor, including lungs and often via the DCD pathway.

## References

[1] Ajdacic-Gross V, Weiss MG, Ring M, Hepp U, Bopp M, Gutzwiller F, Rössler W (2008). Methods of suicide: international suicide patterns derived from the WHO mortality database. Bull World Health Organ.

[2] Large MM, Nielssen OB (2010). Suicide in Australia: meta-analysis of rates and methods of suicide between 1988 and 2007. Med J Aust.

[3] (2021). Australian Bureau of Statistics: 3303.0 - causes of death, Australia, 2017. https://www.abs.gov.au/ausstats/abs@.nsf/Lookup/by%20Subject/3303.0~2017~Main%20Features~Australia%27s%20leading%20causes%20of%20death,%202017~2.

[4] De Leo D, Dwyer J, Firman D, Neulinger K (2003). Trends in hanging and firearm suicide rates in Australia: substitution of method?. Suicide Life Threat Behav.

[5] 3303.0 - Causes of Death, Australia Australia, 2016 2016 (2021). Australian Bureau of Statistics: 3303.0 - causes of death, Australia, 2016. 2021203303020162020.

[6] Hunter E, Harvey D (2002). Indigenous suicide in Australia, New Zealand, Canada, and the United States. Emerg Med (Fremantle).

[7] Trotter P, Robb M, Summers D, Watson C, Neuberger J, Bradley JA (2017). Transplant outcomes in recipients of organs from donors that died from primary hypoxia: the UK experience. Transpl Int.

[8] Mohite PN, Patil NP, Sabashnikov A (2015). "Hanging donors": are we still skeptical about the lungs?. Transplant Proc.

[9] (2021). Australian Bureau of Statistics: 3303.0 - causes of death, Australia, 2015. http://www.abs.gov.au/AUSSTATS/abs.

[10] Thomas M, Klapdor M (2021). Thomas M, Klapdor M: The future of organ donation in Australia: moving beyond the 'gift of life'. Research paper no. 11 2008-09. Parliament of Australia. Research Paper no.

[11] Aufderheide TP, Aprahamian C, Mateer JR (1994). Emergency airway management in hanging victims. Ann Emerg Med.

[12] Adams N (1999). Near hanging. Emerg Med Australas.

[13] (2021). DonateLife Agency, Organ and Tissue Authority, Australian Governement: Our data. (2021). Accessed: October 19. https://www.donatelife.gov.au/about-us/strategy-and-performance/our-data.

[14] (2021). Department of Health, Australian Goverment: The Australian Organ Donor Register. http://www.health.gov.au/internet/main/publishing.nsf/Content/health-organ-aodr.htm.

[15] Figueiredo FM, Capaverde FB, Londero GG (2007). Organ donation in suicides. Transplant Proc.

[16] Ananiadou O, Schmack B, Zych B (2018). Suicidal hanging donors for lung transplantation: Is this chapter still closed? Midterm experience from a single center in United Kingdom. Medicine (Baltimore).

[17] Whitson BA, Hertz MI, Kelly RF, Higgins RS, Kilic A, Shumway SJ, D'Cunha J (2014). Use of the donor lung after asphyxiation or drowning: effect on lung transplant recipients. Ann Thorac Surg.

[18] Renard R, Le Houerou T, Puyo P (2016). Does hanging donors be really marginal for lung transplantation?. J Heart Lung Transplant.

[19] Hoti E, Levesque E, Sebagh M, Heneghan HM, Khalfallah M, Castaing D, Azoulay D (2014). Liver transplantation with grafts from donors who die from suicide by hanging: a matched cohort study. Transplantation.

[20] Nikolić S, Zivković V (2014). Cervical spine injuries in suicidal hanging without a long-drop--patterns and possible underlying mechanisms of injury: an autopsy study. Forensic Sci Med Pathol.

[21] Kumar M, Mandhyan R, Shukla U, Kumar A, Rautela RS (2009). Delayed pulmonary oedema following attempted suicidal hanging-a case report. Indian J Anaesth.

[22] Raj V, Bhatnagar V (2009). Post obstructive pulmonary edema following attempted suicide by hanging. Med J Armed Forces India.

